# Multiclonal human origin and global expansion of an endemic bacterial pathogen of livestock

**DOI:** 10.1073/pnas.2211217119

**Published:** 2022-12-05

**Authors:** Gonzalo Yebra, Joshua D. Harling-Lee, Samantha Lycett, Frank M. Aarestrup, Gunhild Larsen, Lina M. Cavaco, Keun Seok Seo, Sam Abraham, Jacqueline M. Norris, Tracy Schmidt, Marthie M. Ehlers, Daniel O. Sordelli, Fernanda R. Buzzola, Wondwossen A. Gebreyes, Juliano L. Gonçalves, Marcos V. dos Santos, Zunita Zakaria, Vera L. M. Rall, Orla M. Keane, Dagmara A. Niedziela, Gavin K. Paterson, Mark A. Holmes, Tom C. Freeman, J. Ross Fitzgerald

**Affiliations:** ^a^The Roslin Institute, University of Edinburgh, Edinburgh EH25 9RG, United Kingdom; ^b^The National Food Institute, Technical University of Denmark, 2800 Kongens Lyngby, Denmark; ^c^Department for Bacteria, Parasites and Fungi, Reference Laboratory for Antimicrobial Resistance, Statens Serum Institut, 2300 Copenhagen, Denmark; ^d^Department of Basic Sciences, College of Veterinary Medicine, Mississippi State University, Starkville, MS 39762; ^e^College of Science Health, Engineering and Education, Antimicrobial Resistance and Infectious Diseases Laboratory, Murdoch University, Murdoch, WA 6150, Australia; ^f^Sydney School of Veterinary Science, University of Sydney, Sydney, NSW 2050, Australia; ^g^Department of Medical Microbiology, University of Pretoria, Pretoria 0084, South Africa; ^h^Department of Medical Microbiology, Tshwane Academic Division, National Health Laboratory Service, Pretoria 0084, South Africa; ^i^Instituto de Investigaciones en Microbiología y Parasitología Médica, University of Buenos Aires-CONICET, C1121 ABG Buenos Aires, Argentina; ^j^Molecular Epidemiology, College of Veterinary Medicine, Ohio State University, Columbus, OH 43210; ^k^Department of Large Animal Clinical Sciences, College of Veterinary Medicine, Michigan State University, East Lansing, MI 48824; ^l^Department of Nutrition and Animal Production, School of Veterinary Medicine and Animal Sciences, University of São Paulo, Pirassununga, SP, 13635-900, Brazil; ^m^Institute of Bioscience, Universiti Putra Malaysia, 43400 Serdang, Selangor, Malaysia; ^n^Department of Microbiology and Immunology, Institute of Biosciences, São Paulo State University, Botucatu, SP, 18618-970, Brazil; ^o^Animal & Bioscience Department, Teagasc, Grange, Dunsany, Co. Meath C15 PW93, Ireland; ^p^The Royal (Dick) School of Veterinary Studies, University of Edinburgh, Edinburgh EH25 9RG, United Kingdom; ^q^Department of Veterinary Medicine, University of Cambridge, Cambridge CB3 0ES, United Kingdom; ^r^Janssen Immunology, Spring House, PA 19002

**Keywords:** *Staphylococcus aureus*, population genomics, phylodynamics, agriculture, host adaptation

## Abstract

Most new pathogens of humans and animals arise via switching events from distinct host species. However, our understanding of the evolutionary and ecological drivers of successful host adaptation, expansion, and dissemination are limited. *Staphylococcus aureus* is a major bacterial pathogen of humans and a leading cause of mastitis in dairy cows worldwide. Here we trace the evolutionary history of bovine *S. aureus* using a global dataset of 10,254 *S. aureus* genomes including 1,896 bovine isolates from 32 countries in 6 continents. We identified 7 major contemporary endemic clones of *S. aureus* causing bovine mastitis around the world and traced them back to 4 independent host-jump events from humans that occurred up to 2,500 y ago. Individual clones emerged and underwent clonal expansion from the mid-19th to late 20th century coinciding with the commercialization and industrialization of dairy farming, and older lineages have become globally distributed via established cattle trade links. Importantly, we identified lineage-dependent differences in the frequency of host transmission events between humans and cows in both directions revealing high risk clones threatening veterinary and human health. Finally, pangenome network analysis revealed that some bovine *S. aureus* lineages contained distinct sets of bovine-associated genes, consistent with multiple trajectories to host adaptation via gene acquisition. Taken together, we have dissected the evolutionary history of a major endemic pathogen of livestock providing a comprehensive temporal, geographic, and gene-level perspective of its remarkable success.

## Significance

The study uses thousands of bacterial genome sequences to comprehensively dissect the evolutionary history of a leading bacterial pathogen of livestock. Remarkably, contemporary bovine mastitis infections due to *Staphylococcus aureus* can be traced back to historic host switch events that originated in humans up to thousands of years ago. The host jumps were followed by host adaptation by acquisition of different gene combinations followed by global dissemination via established cattle trade links. Our work reveals high-risk clones that undergo more frequent cross-species transmission and which may therefore represent greater threat to public or animal health.

The emergence of new pathogens typically arises through host-jump events and is a major threat to public health and food security (1). The domestication of animals and the expansion of agriculture in the Neolithic era increased the opportunities for the zoonotic and anthroponotic transmission of pathogens (2). Subsequent intensification of farming, industrialization, and globalization have increased the likelihood of successful expansion and dissemination of new pathogenic clones. However, our understanding of the evolutionary history of the major bacterial pathogens affecting livestock is very limited. In order to mitigate the emergence of new pathogens, or design novel interventions to limit spread, it is imperative that we understand the evolutionary and ecological drivers for the success of existing pathogens that have originated via host-switching events.

*S. aureus* is a multihost bacterial species and a major pathogen of humans and livestock. In particular, *S. aureus* is a leading cause of bovine mastitis resulting in huge economic losses to the global dairy industry (3). In addition, bovine *S. aureus* is recognized as an emergent zoonotic threat (4), but the relative frequency of human infections caused by different clones of bovine *S. aureus* has not been examined to date. Previously, we demonstrated that the evolution of *S. aureus* has involved host-switching events between humans and domesticated animals in both directions leading to the emergence of clones circulating in human and livestock populations (5). In addition, we identified gene acquisition as a major driver for the adaptation of *S. aureus* to a new host species after a host-switch event. However, the limited number of isolates from livestock sources included in the study was insufficient to support analysis of the origin, clonal expansion, and global spread of contemporary livestock clones.

Numerous studies have employed whole genome sequencing to explore the diversity of *S. aureus* from dairy cattle but they have tended to include a limited number of isolates from geographically restricted regions (6–15). In order to address this gap in understanding, we established a genome sequence dataset of 10,254 *S. aureus* genomes including 1,896 bovine isolates from 32 countries in 6 continents to carry out a comprehensive phylodynamic and accessory genome network analysis. We provide broad insights into the evolutionary origins of bovine *S. aureus*, including the key impact of human activities, and reveal the adaptive and geographical trajectories that have driven its global success.

## Results and Discussion

### Population Genetic Analysis of Bovine *S. aureus* Reveals Global and Region-Specific Clones.

**Fig. 1.**
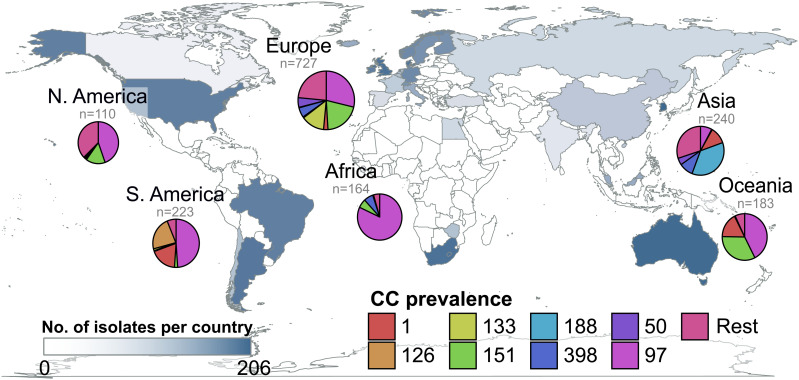
Global distribution of bovine *S. aureus* isolates examined in the study according to CC. The map shows the sampling locations of the 1,647 bovine isolates with available data among the 1,896 total bovine isolates collected. The blue color scale filling each country represents the number of bovine *S. aureus* genomes included in the analyses (log scale). Pie charts indicate the distribution of these genomes per CC in each of the continents represented.

In order to examine the global diversity and distribution of *S. aureus* associated with bovine mastitis, we carried out whole-genome sequencing of 1,034 isolates to add to 862 publicly available sequences resulting in a dataset of 1,896 genomes from cows in 32 different countries across all continents (Fig. 1). Newly sequenced isolates were selected to represent the known geographic and genetic diversity of bovine *S. aureus* including previously under-represented geographical areas (Fig. 1). Genome-based analysis of multilocus sequence type (MLST)-defined sequence types (STs) and clonal complexes (CCs) revealed 209 STs belonging to 53 different CCs among the 1,896 bovine *S. aureus* isolates examined. CC97 was the most prevalent clone globally (32.6%) and in each continent (range of 81.9% in Africa to 28.7% in Europe) except Asia, where CC188 was the most common (36.3%) (Fig. 1). CC151 was also globally distributed (14.5% of all bovine *S. aureus* isolates), with highest prevalence in Europe (20.3%), Oceania (32.8%), and North America (15.5%). Other CCs had a more uneven distribution with several restricted to a single continent or subcontinental region including: CC133, CC522, CC136, and CC130 in Europe (13.3%, 1.7%, 1.4% and 1.3% of the isolates in the region, respectively), CC126 in South America (22.4%), CC350 in North America (9.1%), and CC188 which was exclusive to Asia (36.3%). As indicated in Fig. 1, while our dataset is representative of all continents in the world, some continents such as Africa are sparsely represented relative to others such as Europe and North America. Nonetheless, these data provide an extensive analysis of the global distribution of *S. aureus* STs highlighting major differences in the geographical reach of distinct clones.

### Contemporary Endemic Bovine *S. aureus* Clones Originated from Four Independent Host-Jump Events from Humans during the Last 2,500 y.

**Fig. 2.**
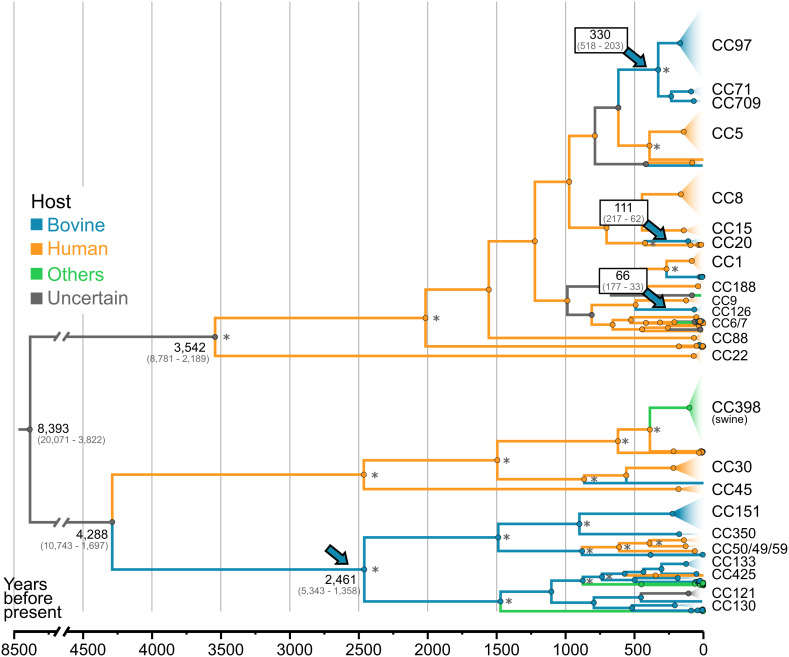
Contemporary bovine *S. aureus* originated from human-to-bovine host switches that occurred in the last 2,500 y. Time-scaled tree of the global, bovine-enriched *S. aureus* dataset (n = 3,915) with host ancestral reconstruction**.** The maximum-likelihood tree was generated with IQ-Tree2, dated using BactDating, and host ancestral states were inferred using SIMMAP. Branches are colored according to the host yielding the highest probability. Main CCs are collapsed in triangles and colored according to their reconstructed original host. Nodes supported by 100% bootstrap are indicated by an asterisk. Key nodes are annotated with their predicted most recent common ancestors (in years before present). Arrows point to the main evolutionary jumps into bovine population.

To examine the relatedness of bovine to nonbovine (human and other animals) *S. aureus* strains and to trace the evolutionary history of bovine *S. aureus*, we generated a whole-genome sequence dataset of 10,254 genomes including 8,361 from human (n = 7,144; 69.7%), porcine (n = 395; 3.9%), ovine (n = 37; 0.4%), avian (n = 385; 3.8%), rodent (n = 356; 3.5%), and other (n = 41; 0.4%) sources to add to the 1,896 (18.5%) of bovine origin (*SI Appendix*, Fig. S1). To provide a dataset that was amenable to time-scaled analysis, among genomes with available sampling dates (n = 9,151), we included all nonredundant bovine *S. aureus* genomes and representatives from all CCs that included >20 isolates (~0.2%) among the nonbovine isolates in the global dataset creating a global, bovine-enriched dataset (1,614 bovine and 2,301 nonbovine) as described in the *Methods* and represented in *SI Appendix*, Fig. S1. A time-scaled phylogeny was reconstructed, whose root was dated at 8,393 y before present (YBP) (CI: 20,071-3,822), with an evolutionary rate of 2.5 (CI: 2.4–2.7) × 10^−6^ substitutions per site per year (s/s/y) consistent with previous estimations of the evolutionary rate for *S. aureus* (16, 17). To examine the frequencies of host species transitions, a reconstruction of ancestral host states across all tree branches was performed considering three states: bovine, human, and other hosts (*SI Appendix*, Table S1). The analysis indicates that humans are the major source of transitions (including host-switch and spillover events) into bovine and nonbovine host species. In addition, cows are a source of *S. aureus* host-switching events into humans highlighting the zoonotic potential of bovine *S. aureus* (Fig. 2).

The ancestral host state for the *S. aureus* species tree was uncertain (each host state was equally likely), but we traced the evolution of the *S. aureus* clones responsible for contemporary bovine mastitis infections to four historical, independent host jumps from humans into cows (Fig. 2). Of the four host jumps, two led to the subsequent emergence of 12 distinct CCs, whereas the other two each led to the emergence of a single CC (Fig. 2). The first of the host jumps occurred about 2,461 YBP [CI: 5343–1358] by the ancestor of contemporary bovine CCs CC151, CC350, CC133, and CC50, and the multihost associated CC49 (Fig. 2). Importantly, a subsequent host jump back into humans led to the emergence of the major epidemic multidrug-resistant ST59 clone that is currently a leading cause of human morbidity and mortality in East Asia (18, 19). Similarly, a host jump back into humans led to the emergence of CC121, a global human clone associated with skin and soft tissue infections (20, 21). A second human-to-bovine jump (Most Recent Common Ancestor [MRCA] = 330 YBP [CI: 518–203]) involved the progenitor of the contemporary bovine *S. aureus* CC97, which has subsequently undergone global expansion to become the most prevalent (36%) clone among our global collection of isolates (Fig. 1). CC71 and CC709 have diverged from CC97 via large-scale recombination events (22), and while CC71 is widely distributed in Europe and the US, CC709 was only identified in Argentina. The more recent third and fourth host jumps led to the bovine CC20 clone found mainly in Asia and in Europe (MRCA = 111 YBP [CI: 216.88–61.98]), and CC126, identified predominantly in Brazil (MRCA = 66 YBP [CI: 177.17–33.46]). Taken together, these data reveal the origins of contemporary bovine *S. aureus* in human to bovine host-switching events that occurred at different timepoints during the last 2,500 y.

### Contemporary Clones of Bovine *S. aureus* Emerged and Expanded in the Second Half of the Nineteenth and Early Twentieth Century.

**Fig. 3.**
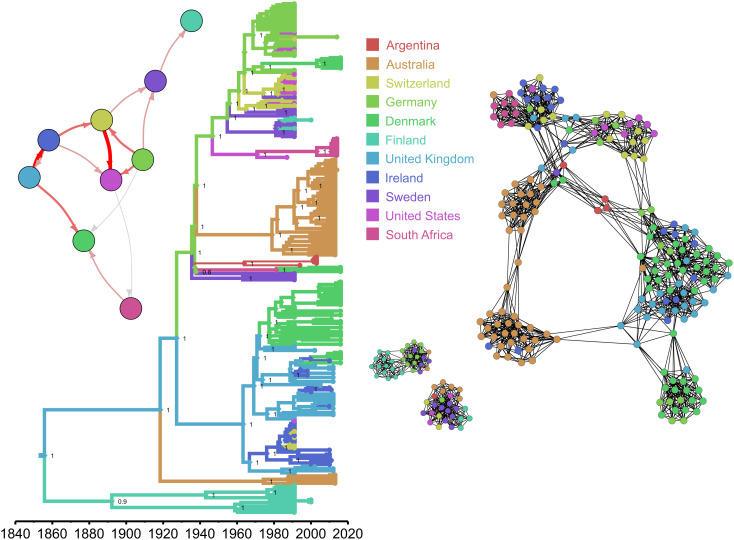
Phylogeographic analysis of the bovine host-restricted *S. aureus* CC151 based on core and accessory genome. Left: Bayesian time-stamped tree from a core genome alignment (1,645,057 bp, of which 14,665 bp were variable sites) of CC151 genome sequences. Branches colored according to the reconstructed locations in the discrete trait analysis. *Inset*: graphic summary of migrations between countries, in which the thickness of arrows is proportional to the number of migration events inferred. Right: Network or accessory genome of the same CC151 sequences (based on 483 accessory genes, defined as genes in more than 1 genome, and not in all genomes). Node colors correspond to sampling locations in both the tree and the network.

**Fig. 4.**
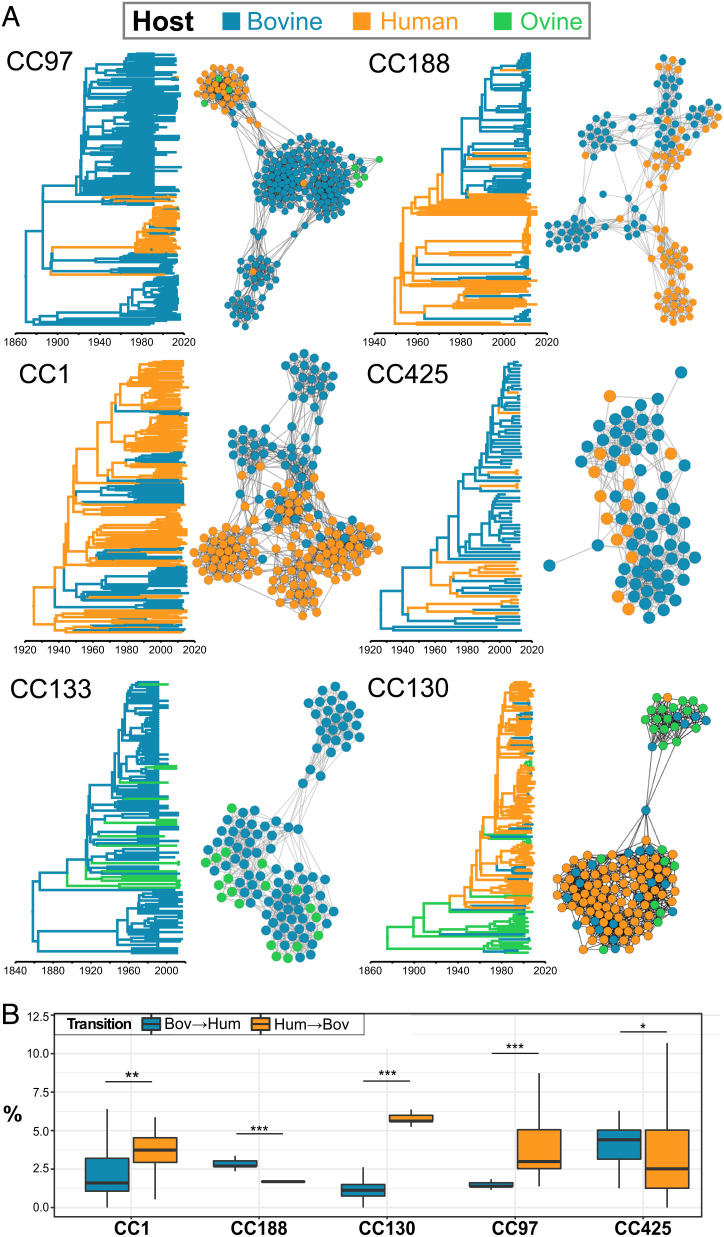
Lineage-dependent differences in the frequency of host transitions reveal high-risk *S. aureus* clones. (*A*) CC-specific analysis of ancestral host reconstruction**.** Analyses for each CC include a time-stamped phylogenetic tree generated using the MASCOT model in *BEAST2* to reconstruct ancestral host state; and a network representing the accessory genome of the same samples. Each node represents a genome, and edges are weighted as the similarity between two genomes. The number of *S. aureus* genomes used for each analysis was CCC151: 246 bovine; CC97: 175 bovine, 35 human, 8 ovine; CC188: 86 bovine, 63 human; CC1: 67 bovine, 121 human; CC425: 65 bovine, 15 human; CC133: 98 bovine, 19 ovine; CC130: 23 bovine, 86 human, 25 ovine (*SI Appendix*, Fig. S1 and Dataset S1). (*B*) Percentage of *S. aureus* transitions between bovine and human host species over the total of transitions per tree in the *BEAST2* tree posterior distribution. CC1 and CC188 are CCs with human origins, CC97 and CC425 with bovine origins, CC130 with ovine origin. CC133 not shown as no human isolates included in the analyses.

In order to further examine the geographical dissemination and host species phylodynamics of contemporary bovine *S. aureus*, the seven most predominant bovine *S. aureus* CCs in our collection (for each of which we had at least 150 genome sequences) were selected for high-resolution phylodynamic analyses using a structured coalescence model in *BEAST2* (23). We employed this model as it provides an ancestral trait reconstruction that is much more robust to sampling bias than the classical discrete trait analyses, recognizing the uneven nature of our bovine *S. aureus* sample set with regard to host species and geography (24) (*SI Appendix*, Table S3). Of the 7 CCs examined, 5 were inferred to have an MRCA that existed in ruminants (CC97, CC151, CC133, and CC425 in cattle and CC130 in sheep) and emerged in the mid-second half of the 19th century. CC151 is a globally distributed bovine-exclusive lineage that exhibited a strong phylogeographic structure with subclades often correlated with specific countries or regions (Fig. 3). CC97 is also globally widespread in dairy cows, but is characterized by two independent host switches back into humans as previously reported (22). Our analysis revealed that one of the CC97 bovine-to-human host switches which occurred about 48 y ago (MRCA = 1974 [1969–1978]) subsequently led to global dissemination via at least 12 countries in 6 continents (Europe, North and South America, Asia, Oceania, and Africa) with several instances of spillovers back into cattle (Fig. 4 and *SI Appendix*, Fig. S2). In contrast, the other CC97 host switch into humans (MRCA = 1972 [1959–1983]) led to a clone that has since only been isolated from humans in the UK (*SI Appendix*, Fig. S2). Similarly, CC425 is largely restricted to cattle in the UK (although it has also been reported in European and UK wildlife (25)) with an MRCA that existed about 100 y ago in a bovine host but has been marked by several host switches and spillover events into human populations (Fig. 4). CC133 is only found in cattle and sheep, and despite being the most prevalent ovine-associated lineage (26), our analyses reveals a likely origin in cattle with multiple introductions into sheep during its history. Phylogeographic analysis of CC133 reveals many transitions between Northern European countries, presumably reflecting close livestock trade links in Scandinavia (*SI Appendix*, Fig. S3). The contemporary clone CC130 is a result of the earliest host jump from humans into ruminants with sheep being the likely ancestral host of the CC before a subsequent host switch into humans (MRCA = 1914 [1889–1938]) followed by spread across human populations in Europe. Contemporary bovine isolates of CC130 represent numerous spillover events from either humans or sheep, highlighting the capacity of CC130 for multihost species transitions (Fig. 4 and *SI Appendix*, Fig. S4).

In contrast, of the 7 major bovine *S. aureus* CCs analyzed, the two that had an MRCA of human origin (CC1 and CC188) emerged later, in mid-to-late 20th century (Fig. 4). CC1 is one of the most important community-associated human lineages disseminated worldwide (27). Remarkably, our analyses demonstrate numerous human-to-bovine host jumps that occurred independently in CC1 in different regions in the world followed by geographically limited expansions, in Australia (MRCA = 1971 [1955–1987]), Australia/Malaysia (MRCA = 1989 ([1979–1999]), Argentina/Brazil (MRCA = 1969 [1958–1981]), and Korea (MRCA = 1989 [1979–1999]) (Fig. 4 and *SI Appendix*, Fig. S5). Finally, CC188 originated in humans but has subsequently been associated with at least 3 host jumps into cattle in Asia (MRCAs = 1983 [1974–1991], 2007 [2005–2009], and 2007 [2004–2009]) (Fig. 4 and *SI Appendix*, Fig. S6). These data reveal *S. aureus* human lineages with the capacity to undergo regular host switches into dairy cows and which therefore represent high-risk clones, which should be the target of ongoing surveillance in human and dairy cow populations. Recently, *Campylobacter jejuni* was shown to have expanded in cattle during a similar era (20th century), coinciding with more intensive farming and the increase in cattle population size (28).

Taken together, these analyses reveal the distinct geographical and host species associations observed for each of the major *S. aureus* clones associated with dairy cows. Importantly, some clones exhibit a greater propensity to undergo shifts in host species ecology, including the capacity for zoonoses and/or transmission in human populations.

### Identification of Routes of Global Dissemination via International Trade Links.

**Fig. 5.**
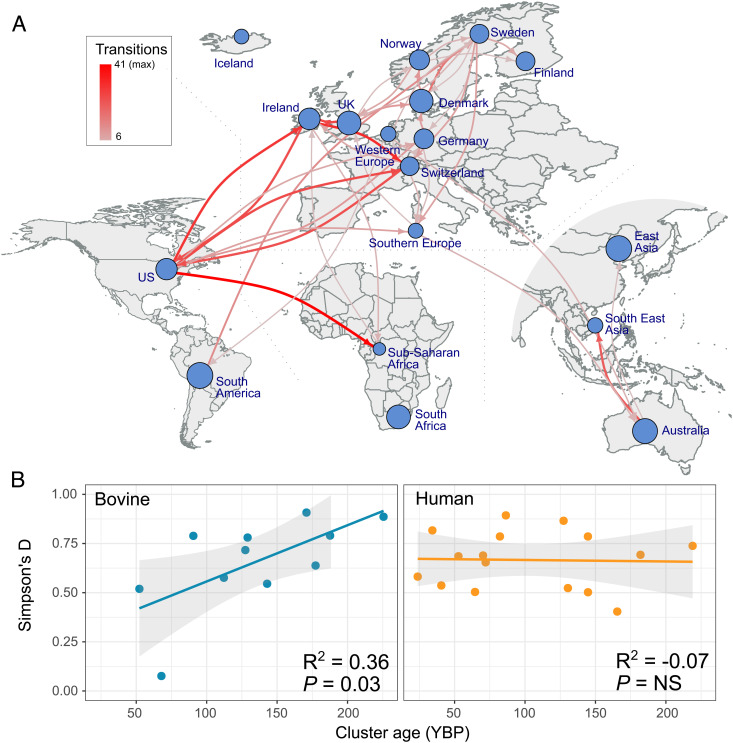
Routes of international dissemination for bovine *S. aureus* revealed by ancestral trait analysis. This analysis was performed on the bovine-enriched *S. aureus* dataset (n = 3,915) which included 1,614 bovine assemblies with known sampling time and locations. (*A*) Schematic worldwide map showing inferred location transitions within the bovine population. Nodes represent each location (individual countries or groups of countries) included in the analysis. Arrows represent estimated, directional migrations (i.e., location state changes) between locations. Arrows width and color are proportional to the estimated amount of migrations. (*B*) Correlation between age (in years before present) of the CCs of ancestral bovine and human origins and the Simpson’s D diversity index calculated from the number of countries from which they were identified in our dataset, showing a statistically significant positive correlation.

Considering the wide spatial distribution of bovine *S. aureus* STs identified in the current study, we wished to examine the main routes of transmission of *S. aureus* among global dairy cow populations. Accordingly, ancestral trait reconstruction was applied to the global dataset of bovine *S. aureus* sequences based on sampling location data. Bovine *S. aureus* sequences were labeled according to their country of isolation, and isolates from countries less well represented were grouped into larger geographical regions. Based on this analysis, a high number of statistically supported migrations were identified, among the most frequent of which were bidirectional migrations between North America (USA/Canada) and European countries such as Ireland, Switzerland, and the UK (Fig. 5 and *SI Appendix*, Table S2). In addition, a highly supported migration was identified between North America and sub-Saharan Africa (Zimbabwe, Sierra Leone, and Tanzania). Conversely, some well-represented locations such as South America, East Asia, and Australia were involved in relatively few migration events consistent with restricted international movement of bovine *S. aureus* to or from those countries. Of note, Australia was identified only as a source of introductions to South East Asia (Malaysia/Thailand), consistent with their global proximity and with Australia’s strict regulations regarding international trade of livestock (29). Finally, we investigated the hypothesis that the extent of global dissemination of individual clones reflected the time since those clones emerged. We identified a positive correlation between the age of each bovine CC or subclade (estimated from the time-scaled phylogeny as described below) and the Simpson D diversity index of the number of countries in which it had been isolated (R^2^ = 0.36, *P* = 0.03) (Fig. 5). Although there are clear gaps in global sampling of bovine *S. aureus* as highlighted previously, the data suggest that older bovine *S. aureus* clones have a greater geographical reach because they have had more time to spread, presumably via the international trade of cattle. In contrast, there was no significant correlation between the age of human *S. aureus* clones and the diversity of countries from which they were isolated. This suggests that human strains very rapidly disseminate around the world after their emergence, presumably due to the frequent migration of human hosts via air travel (Fig. 5). Our analysis is limited by the gaps in global sampling, and broader inclusion of samples from different geographical regions would provide greater resolution of transmission networks. Overall, these data reveal routes of global migration of bovine *S. aureus* and identify distinct spatial dynamics in comparison with human *S. aureus* clones.

### Identification of High-Risk *S. aureus* Clones with Enhanced Host-Switching or Zoonotic Potential.

The reconstruction of ancestral host states for the global dataset revealed that the dairy bovine population is predominantly a sink, and human population a source of host-switching events. However, phylodynamic analyses of individual CCs revealed different clone-dependent patterns of transient spillover (i.e., host jump between host species without onward transmission to other individuals), or host switching (host jump between species with onward transmission). For example, CC188 and CC1 exhibited host-specific clusters of isolates indicative of host switching, while others had differing frequencies of spillover events (Fig. 4*B*). CC97 was characterized by host switching into humans and spillover events in both directions between humans and dairy cows, and analysis of CC130 revealed that it likely originated in sheep before expansion in human populations, with numerous independent spillovers from humans into dairy cows. Conversely, CC425 is predominantly bovine-associated but has been characterized by many spillover events into humans, indicating a higher risk of zoonoses compared with other lineages (Fig. 4). In contrast, many clonal lineages appear to be largely host restricted with very limited capacity to transmit between host species. In particular, CC151 is a major global lineage of bovine *S. aureus* but has not to date been isolated from humans. Similarly, major global human lineages such as CC22 and CC5 are rarely if ever identified in bovine samples. Furthermore, we have identified CC188 to have contributed to multiple independent human to bovine host jumps in Asia, and it is noteworthy that no such jumps have been identified by CC188 in other parts of the world to date, despite being widespread. This may suggest differences in farming practices or ecology that influence interhost species transmission in different parts of the world. Taken together, these data reveal intrinsic differences between different *S. aureus* clones in their capacity to switch between host species and identifies specific clones associated with more frequent zoonotic episodes. The basis for these distinct tropisms may reflect genetic or ecological differences that require investigation.

### Bovine *S. aureus* Clones Contain Distinct Gene Combinations that Underpin Host Adaptation.

**Fig. 6.**
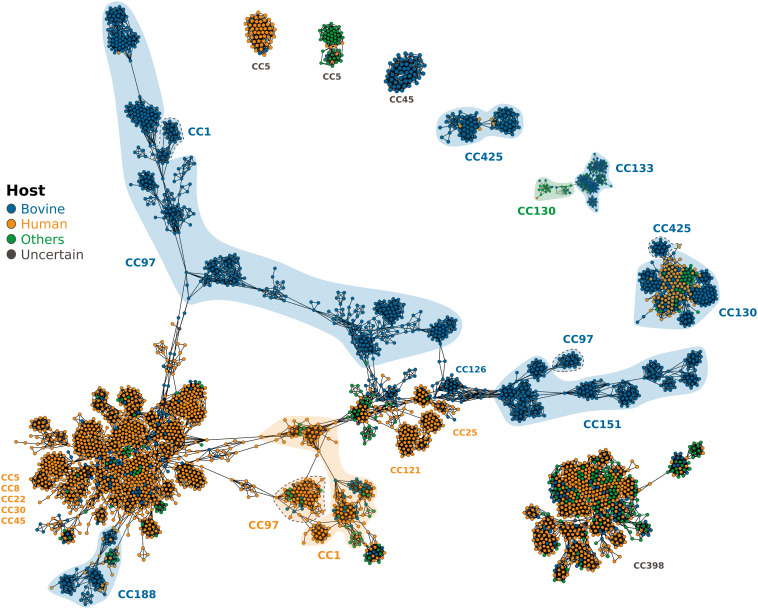
Network analysis of the accessory genome reveals host species and CC-dependent gene combinations. Pairwise similarity network of 4,841 *S. aureus* isolates based on shared accessory genes. For host species association see *SI Appendix*, Fig S1). Each node represents a genome, and edges are weighted as the similarity between two genomes, with lineage-dependent accessory genes removed. Similarity was calculated using the Jaccard Index, and the network visualized within Graphia using a force-directed 2D layout. A k-NN (k = 10) algorithm was applied to reduce edge density, and components of less than 50 nodes removed. The network is colored by the host species, and important CCs are highlighted, colored by the primary host species of the CC or subcluster (dotted gray lines).

Previously, we reported that gene acquisition is associated with *S. aureus* adaptation to a new host species after a host switch (5). The large genome dataset compiled for the current study provided an opportunity to comprehensively examine the diversity of the bovine *S. aureus* accessory genome and its relationship with host species and geography. The genome sequence dataset of 4,841 *S. aureus* isolates representing the global and CC-specific datasets was determined to comprise a total of 24,299 genes (defined at the 95% identity threshold). Clustering analysis based on the presence of accessory genes revealed 59 major clusters (MCLi = 1.20), 58 of which were significantly enriched with at least one CC indicating a strong lineage-dependence (Goodman–Kruskal tau (GK-τ) value for correlation of accessory genome clusters with CC of 0.934 (*SI Appendix*, Table S4). Similarly, 57 of 59 clusters were significantly associated with at least one host species, though with a lower GK-τ of 0.494). We next removed all lineage-restricted genes (defined as those present in >95% of isolates from one or more lineages) and continued to observe a correlation with CC, albeit at a reduced level (GK-τ = 0.828) and an increased correlation with host species (GK-τ = 0.61) (MCLi = 1.20)). In particular, we observe several instances of CCs that exhibit distinct host-specific accessory genome clusters, including CC1 and CC97, (human and bovine clusters), and CC130, human and ovine clusters (Fig. 6).

Focusing on individual CCs of bovine-associated *S. aureus* revealed lineage-dependent correlates of accessory genome with host species and geographic location (Figs. 3 and 4 and *SI Appendix*, Figs. S2–S6 and Table S4). For example, the multihost-associated CC97, CC1, and CC188 have distinct accessory clusters that correlated strongly with host species. In contrast, CC425 isolates had accessory genomes that clustered independently of host species. This supports the idea that human CC425 infections are due to spillover events from cattle with limited evidence of host adaptation and onward transmission (Fig. 4). Similarly, analysis of CC133 associated with bovine and ovine mastitis infections did not reveal any host-specific accessory gene clusters. Allied to the phylogenetic analysis of CC133 that revealed interspersal of cattle and sheep isolates across the phylogeny, these findings are consistent with CC133 adaptation to the intramammary milieu rather than to distinct ruminant host species.

We also tested the hypothesis that accessory gene content may be influenced by geographic location, perhaps reflecting distinct gene pool reservoirs in different ecological settings around the world. Of the 7 CCs examined, CC151, CC188, and CC425 exhibited a signal of location association in accessory network analysis (*SI Appendix*, Table S4). However, overall, the accessory genome was not strongly affected by geographical location (*SI Appendix*, *Materials*). These findings imply relatively stable accessory gene combinations that are maintained even after geographic migration events.

### Identification of Accessory Genes Associated with Bovine Host Adaptation.

To identify genes significantly enriched in bovine *S. aureus* and underrepresented among nonbovine isolates, consistent with a role in host adaptation, adjusted Fisher’s tests were carried out. Analysis of the global *S. aureus* dataset revealed 841 genes enriched in bovine compared with non-bovine *S. aureus* including 458 (54.46%) that are lineage independent (Dataset S2). We also examined the host-dependent distribution of genes in individual CCs that had a multihost species ecology including CC1, CC97, and CC188, and identified 155 genes enriched in bovine *S. aureus* isolates; 73 in CC1, 52 in CC97, and 30 in CC188. Of note, only a very limited number of bovine-specific genes were significantly enriched in multiple CCs; 14 in CC1 and CC97, and 2 in CC188 and CC1, of which 1 is also shared with CC97 (Dataset S3). A total of 71% of genes identified to be bovine host associated are of unassignable or unknown functional categories. For those that could be assigned (242 of 841; 28.78%), the most common COG categories were M (20.25%; Cell wall, membrane biogenesis), K (20.25%; Transcription), and L (18.18%; Replication, Recombination and Repair) (Datasets S2 and S3). Taken together, the lineage dependence of the accessory genome and the limited sharing of bovine-associated genes between CCs strongly support the idea that distinct combinations of genes acquired by different lineages can underpin the adaptation to a bovine host ecology.

We also examined the distribution of methicillin-resistant *S. aureus* (MRSA) among the diversity of bovine isolates by identifying *mecA* and *mecC* genes that encode alternative penicillin-binding proteins mediating resistance to all ß-lactam antibiotics. *mecA* was present in 177 (8.87%) of bovine isolates, predominantly in CC398, CC5, and CC97 (*SI Appendix*, Table S5). In CC398 and CC97, all bovine *mecA* carriage is associated with spillover events originating in humans or swine. *mecC* was identified in 216 (10.82%) bovine isolates, almost exclusively in CC130 (n = 109) and CC425 (n = 101) (*SI Appendix*, Table S6), consistent with previous observations made (30). The basis for the narrow distribution of *mecC* within a small number of lineages across the species phylogeny is unclear, but a very recent study describes how *mecC* was likely acquired by distinct *S. aureus* lineages associated with hedgehogs prior to the therapeutic use of antibiotics. This was likely due to the resistance it conferred to a beta-lactam antibiotic produced by cocolonizing strains of the dermatophyte *Trichophyton erinacei* (31). These data suggest that the ecology of *S. aureus* strains and the microbiota of their preferred host species has likely impacted on the distribution of the *mecC* gene, and cattle may represent intermediate hosts in the zoonotic transmission of CC130 and CC425 as proposed by Larsen et al (31). The data also indicate that, in contrast to pig farming, MRSA do not frequently emerge in dairy cows possibly due to less intensive farming and distinct antibiotic usage practices. While much of the global dairy industry still involves pasture-based farming, there is a move toward a smaller number of dairy farms with large numbers of animals in some parts of the world such as North America, which may involve increased use of antibiotics for controlling infection (32). It remains to be seen if this trend leads in the future to increased levels of antimicrobial resistance among mastitis pathogens.

**Fig. 7.**
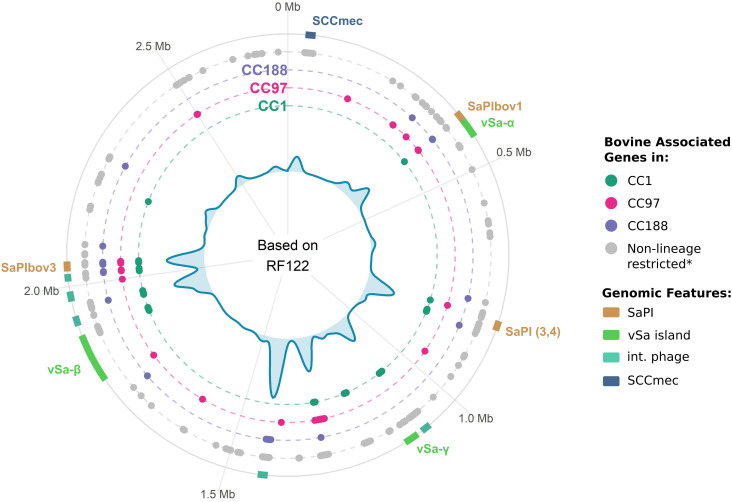
Bovine-specific *S. aureus* genes are associated with phage, SaPIs, and genomic islands*.* Bovine-associated genes were mapped to the bovine reference genome RF122 to model genomic location. The central density plot represents the distribution of all bovine-associated genes identified in this study. Circle dot plots represent the location of bovine associated genes as identified in, from inner-most to outward, CC1 (green), CC97 (pink), CC188 (purple) and the full dataset (gray; *the bovine exclusive lineages CC126 and CC151 core genes are included here). Known genomic features of interest are displayed as blocks on the outermost plot.

Finally, construction of a synteny map for the bovine *S. aureus* pangenome revealed the genomic location for genes enriched in bovine *S. aureus*. We find a non-random genomic distribution of bovine-specific genes, with 223 and 103 bovine-associated genes located in integrated phage elements and *S. aureus* pathogenicity islands (SaPIs), respectively, along with significant enrichment in genomic islands such as SCCmec, vSaα, and vSaβ (Fig. 7 and *SI Appendix*, Fig. S7). Taken together, the pangenome analysis of bovine *S. aureus* has revealed distinct gene combinations that underpin adaptation to a bovine host ecology by different clones, associated mainly with phage and SaPI-mediated gene acquisition. The large numbers of bovine-associated genes of unknown function suggest an incomplete understanding of the mechanisms involved in bovine host adaptation.

### Concluding Comments.

Taken together, we have traced the evolutionary history of a leading cause of the most important disease impacting the global dairy industry. Our findings highlight the impact of human activities from domestication of cattle to industrialization and globalization of farming on the emergence and dissemination of bovine *S. aureus*. Pangenome analysis identified different host-adaptive evolutionary trajectories for the major bovine *S. aureus* clones associated with acquisition of distinct lineage-dependent gene combinations. Importantly, differences in the frequency of host species transmission events between different clones was discovered, and the identification of high-risk clones with greater propensity for human zoonoses may facilitate targeted mitigation of future public health threats.

## Methods

### Limitations of the Sample Dataset.

This is a dataset of genome sequences from isolates collected in an array of studies around the world. As such, it is limited to what has been previously sampled and was made available to us. Clearly, for many countries we do not have any samples, and unsampled reservoirs or intermediate hosts cannot be ruled out. In particular, we have limited numbers of samples from Africa and Asia. However, we have compiled a large dataset of 10,254 genomes of which 1,896 were of bovine origin in 32 countries spanning 6 continents. In order to limit the effects of sample bias, for the time-scaled phylogenies with traits, we subsampled sequence clusters with shared location/host in each tree to avoid redundancy, and used grouped locations (e.g., South East Asia rather than individual countries within SE Asia) with a minimum of 10 sequences per location. In addition, we used a structured coalescent model, that is less affected by sampling bias than traditional discrete trait analysis using BSSVS for inferring the rates between locations or hosts (24), and we report inferred numbers of events (with confidence intervals) rather than relative rates. Finally, we also performed tip permutation to assess the effects of uneven numbers of sequences in categories in *BEAST2* and *BaTS* (33).

### Whole Genome Sequence Dataset.

A collection of 1,896 genome assemblies from bovine *S. aureus* isolates was created (See Dataset S1 for details and accession numbers). It included 1,034 newly generated whole-genome sequences (WGS) of bovine strains from 18 countries throughout the world, using the Nextera XT library prep protocol on a MiSeq platform (Illumina, San Diego, CA, USA). We added 357 previously generated, Illumina-based WGS (5, 34). These sequences were combined with all available *S. aureus* bovine sequences from public repositories (97 assemblies from GenBank and 394 Illumina short read sequences downloaded from the Sequence Read Archive). Short read sequences were adapter trimmed using *Trimmomatic* v0.36 (35) and de novo assembled using *SPAdes* v3.11.1 (36). Genes were annotated using *Prokka* v1.13 (37). All assemblies were typed using *mlst* v2.10 (https://github.com/tseemann/mlst) and STs were grouped into CCs using the *eBURST* algorithm in *PhyloViz* v2.0 (38). We added to this dataset all *S. aureus* RefSeq genomes with known host belonging to the largest nonbovine host categories (i.e., human, swine, avian, ovine, and rodent) as background sequences, which produced a dataset of 10,254 genomes among which 1,896 were bovine.

### Phylodynamic Analyses.

In order to explore the phylodynamics of bovine *S. aureus* in the context of the S. aureus species as a whole, we constructed a global, bovine-enriched *S. aureus* sequence dataset (see *SI Appendix*, Fig. S1 for a schematic explanation of the different datasets used). Starting with those genomes with available sampling dates (n = 9,151, of which 1,614 were bovine) among the full dataset, we included all nonredundant bovine *S. aureus* genomes and representatives from all CCs that included >20 isolates (~0.2%) among the 8,358 nonbovine isolates in the global dataset. This included the 50 genetically closest nonbovine assemblies (expressed in *mash* genetic distances (39)) for each bovine assembly. The final global phylodynamic dataset included 3,915 genomes: 1,614 bovine (41.2%), 1,756 human (44.9%), 244 rodent (6.2%), 190 porcine (4.9%), 60 avian (1.5%), 16 ovine (0.4%), and 35 others (0.9%). A core SNP alignment was constructed using *snippy* and *snippy-core* v1.4 (https://github.com/tseemann/snippy) using the genome of the RF122 isolate (GCA_000009005.1) as reference. Recombinant regions were identified using *Gubbins* v2.3.4 (40) and discarded from the alignment. A maximum-likelihood (ML) tree was constructed with *IQ-TREE2* v2.0.5 (41) using the GTR+G model of nucleotide substitution and performing 1,000 bootstrap replicates.

A Bayesian time-stamped analysis of this tree was performed using the R package *BactDating* (42) in order to fit a molecular clock and estimate dates of all ancestral nodes. Discrete trait reconstruction analyses aimed to infer ancestral states, and state changes across the branches of the phylogeny for host and location were performed using 100 simulations of stochastic character mapping (SIMMAP) (43) as implemented in the R package *phytools* (44). Both symmetric (“SYM”) and asymmetric (“ARD”) models were tested, and the median number and 95% highest posterior density of state changes were recorded.

The seven most abundant bovine-associated lineages were selected for further, in-detail phylodynamic analyses: CC151, CC97, CC1, CC133, CC188, CC425, and CC130. We complemented each of these datasets by identifying in public repositories additional sequences belonging to specific STs within each CC using the R package *staphopia-r* (45). Those with available metadata (sampling date, location, and host) were downloaded from SRA, processed as above and included in the analysis. Due to its computational requirements, BEAST analyses were restricted to a maximum of ~250 genomes per CC-specific dataset. If more were available, they were subsampled by excluding genetically similar genomes isolated in the same locations in similar dates. CC-specific recombination-free core SNP alignments and ML trees were constructed using the same pipeline as above. Temporal signal of each tree was estimated using *TempEst* v1.5.3 (46). For these seven datasets, phylodynamic analyses were performed in *BEAST* v2.6.1 (23) following a structured coalescence-approximation model implemented in the MASCOT package (47), which was coupled with a relaxed molecular clock model and the GTR model of nucleotide substitution. Each was run with two sets of discrete traits, namely sampling location and host. The number of changes between trait states was calculated by counting those cases when the most probable state of a lineage changed between its parent and child node. In order to support a nonrandom distribution of the state change counts, a permutation test in which BEAST was rerun 10 times per dataset and trait by randomly swapping the states associated to each tip. The distributions of state change counts within and across runs were statistically compared using a two‐sample Wilcoxon test as previously demonstrated (48).

### Network Analyses of the Accessory Genome.

Annotated genomes in GFF3 format were used as input to *PIRATE* v1.0.4 (49), and the pangenome identified using default thresholds and settings, and paralogs were split. The *PIRATE* output was adapted for analysis in *Graphia* (50) using the bespoke *GraPPLE* scripts (51). The pangenome at 95% identity was created, and the pairwise Jaccard similarity coefficient calculated between genomes and genes, with JSC > 0.5 retained using *pw_similarity.py*. Networks were filtered in *Graphia* by edge weight and the k-Nearest Neighbours algorithm. Networks for CCs had edges filtered by the JSC (weight < 0.8), and then the k-NN (k = 10) algorithm. Each genome and gene similarity network was clustered using the MCL algorithm (inflation values for individual networks varied depending on structure and size). Syntenic relationships between genes were investigated by transforming the pangenome.edges output from *PIRATE* to a .LAYOUT format (using the *edges_to_layout.py GraPPLE* script under default settings), and the resulting network file visualized using *Graphia*. Enrichment analyses were performed using the in-built Enrichment tool in *Graphia*. Goodman–Kruskals’ tau was calculated with the R package *GoodmanKruskal* v0.0.3 in R v3.5.3.

### Accessory Gene Association Analysis.

Lineage specificity of all genes was determined using *Twilight* (52), with the minimum group size for a CC to be included set as >1% of the total dataset (19 for bovine; 45 for global). Core genes were defined as those present in 95% of genomes, and the rare gene threshold set to 5%. Gene associations to host and location were tested using *Scoary* v1.6.16 (53) with default settings, in both the global and CC-specific datasets; significance was set at a Bonferroni adjusted *P*-value < 0.05, and results from the global dataset were filtered by Specificity value > 80. Functional categories of bovine-enriched accessory genes were predicted using EggNOG-mapper (v2.0) (54), Bakta (v0.5) (57) , and InterProScan (v5.52-86.0) (55). To construct the gene enrichment map, the nearest RF122 and core loci in the synteny network were identified for each enriched gene, and the distance from the core loci used to estimate a position for the gene in RF122. Genomic features were mapped according to a previous study (56). Representative sequences for all genes identified in this study are given in Dataset S5.

## Supplementary Material

Appendix 01 (PDF)

Dataset S01 (XLSX)

Dataset S02 (XLSX)

Dataset S03 (XLSX)

Dataset S04 (XLSX)

Dataset S05 (TXT)

## Data Availability

Raw DNA Illumina sequence data have been deposited in ENA/SRA. Accession codes for these and for the already available data used in this study are provided in (Dataset S1).
