# Supplementary material for: Multiclonal human origin and global expansion of an endemic bacterial pathogen of livestock
Source: Proc Natl Acad Sci U S A. 2022 Dec 5;119(50):e2211217119. doi: 10.1073/pnas.2211217119 (PMC9897428; doi:10.1073/pnas.2211217119)
Supplement: Supplementary file 1 — Appendix 01 (PDF) [file pnas.2211217119.sapp.pdf]

Supplementary Information for

Multiclonal human origin and global expansion of an endemic bacterial pathogen of livestock

Gonzalo Yebra[1]*, Joshua D. Harling-Lee [1]*, Samantha Lycett[1], Frank M. Aarestrup[2], Gunhild Larsen[2], Lina Cavaco[3], Keun Seok Seo[4], Sam Abraham[5], Jacqueline M. Norris[6], Tracy Schmidt[7], Marthie M. Ehlers[7,8], Daniel O. Sordelli[9], Fernanda R. Buzzola[9], Wondwossen A. Gebreyes[10,11], Juliano L. Gonçalves[12], Marcos V. dos Santos[12], Zunita Zakaria[13], Vera L. M. Rall[14], Orla M. Keane[15], Dagmara A. Niedziela[15], Gavin K. Paterson[1,16], Mark A. Holmes[17], Tom C. Freeman[1,18], and J. Ross Fitzgerald[1 †]

(1) The Roslin Institute, University of Edinburgh, Edinburgh, UK; (2) The National Food Institute, Technical University of Denmark, Lyngby, Denmark; (3) Statens Serum Institute, Copenhagen, Denmark; (4) Department of Basic Sciences, College of Veterinary Medicine, Mississippi State University, Starkville, MS, United States; (5) Antimicrobial Resistance and Infectious Diseases Laboratory, College of Science, Health, Engineering and Education, Murdoch University, Murdoch, WA, Australia; (6) Sydney School of Veterinary Science, University of Sydney, Sydney, Australia; (7) Department of Medical Microbiology, University of Pretoria, Pretoria, South Africa; (8) Department of Medical Microbiology, Tshwane Academic Division, National Health Laboratory Service, Pretoria, South Africa; (9) Instituto de Investigaciones en Microbiología y Parasitología Médica, University of Buenos Aires-CONICET, Buenos Aires, Argentina; (10) Molecular Epidemiology, College of Veterinary Medicine, the Ohio State University, Columbus, USA; (11) Department of Large Animal Clinical Sciences, College of Veterinary Medicine, Michigan State University, East Lansing, MI, USA; (12) Department of Nutricion and Animal Production, School of Veterinary Medicine and Animal Sciences, University of São Paulo, Pirassununga, SP, Brazil; (13) Institute of Bioscience, Universiti Putra Malaysia, Serdang, Malaysia; (14) Department of Chemical and Biological Sciences, Institute of Biosciences, São Paulo State University, Botucatu-SP, Brazil, (15) Animal & Bioscience Department, Teagasc, Grange, Dunsany, Co. Meath, Ireland; (16) R(D)SVS, University of Edinburgh, Edinburgh, UK; (17) Department of Veterinary Medicine, University of Cambridge, Cambridge, UK; (18) Janssen Immunology, Spring House, PA, USA.

* These authors contributed equally

† Corresponding author: J. Ross Fitzgerald (ross.fitzgerald@roslin.ed.ac.uk)

This PDF file includes:

Supplementary Materials

Figures S2 to S7

Tables S1 to S6

Supplementary Materials

Accessory Genome & Geographical Analysis

To identify genes significantly enriched in specific geographic locations, we performed adjusted Fisher's tests within each CC dataset. Our primary aim was to identify any genes enriched in the same location across multiple CCs, as this would provide evidence for genes inhabiting a geographic niche. Were this true, we would expect subsequent acquisition of such genes by a foreign CC upon migration into that niche. However, we found just 36 genes positively associated (Bonferroni corrected $p < 0.05$) with the same location in two of the seven CCs, and none in three or more CCs (**SI Dataset 5**). Of those 36, 15 are positively associated with Norwegian CC130 and CC133 isolates. We also identify just 19 genes negatively associated (Bonferroni corrected $p < 0.05$) with a single location; 11 of these are negatively associated with Norwegian CC130 and CC133 isolates (**SI Dataset 5**).

Supplementary Figures

Full Dataset (n=10,254) → Fig.1

Host	#	%
Human	7,144	69.7
Bovine	1,896	18.5
Swine	395	3.9
Avian	385	3.8
Rodent	356	3.5
Ovine	37	0.4
Other	41	0.4
Total	10,254	100.0

Continent	#	%	# Bovine	%
N. America	4,759	46.4	110	5.8
Europe	3,180	31.0	727	38.3
Asia	690	6.7	240	12.7
S. America	520	5.1	223	11.8
Oceania	249	2.4	183	9.7
Africa	208	2.0	164	8.6
Not Available	648	6.3	249	13.1
Total	10,254	100.0	1,896	100.0

Bovine genomes with sampling date (n=1,614)
+
Genetically closest non-bovine genomes with sampling date, including representatives of all non-bovine CCs that take up ≥0.2% of the full dataset (n=2,301)

Bovine-enriched Phylodynamic Dataset (n=3,915) → Figs. 2, 5

Host	#	%
Human	1,756	44.9
Bovine	1,614	41.2
Rodent	244	6.2
Swine	190	4.9
Avian	60	1.5
Ovine	16	0.4
Other	35	0.9
Total	3,915	100.0

Continent	#	%	# Bovine	%
Europe	1,655	42.3	696	43.1
N. America	974	24.9	106	6.6
Asia	514	13.1	240	14.9
S. America	308	7.9	223	13.8
Oceania	208	5.3	183	11.3
Africa	183	4.7	164	10.2
Not available	73	1.9	2	0.1
Total	3,915	100.0	1,614	100.0

Addition of extra genomes from *Staphopia* for the CCs with the most bovine genomes

Seven CC-specific phylodynamic analyses → Figs. 3, 4, S2-6

CC	CC151	CC97	CC188	CC1	CC425	CC133	CC130
Bovine	246	175	86	67	65	98	23
Human	0	35	63	121	15	0	86
Ovine	0	8	0	0	0	19	25
Total	246	218	149	188	80	117	134

Accessory Genome Network Dataset (n=4,841) → Fig. 6

Host	#	%
Human	2,209	45.6
Bovine	1,996	41.2
Rodent	250	5.2
Swine	220	4.5
Avian	73	1.5
Ovine	43	0.9
Other	50	1.0
Total	4,841	100.0

Continent	#	%	# Bovine	%
Europe	1,738	35.9	830	41.6
N. America	1,100	22.7	240	12.0
Asia	518	10.7	222	11.1
S. America	319	6.6	185	9.3
Oceania	216	4.5	162	8.1
Africa	192	4.0	91	4.6
Not available	758	15.7	266	13.3
Total	4,841	100.0	1,996	100.0

Fig. S1: **Schematic description of the datasets used in this study and the relationships between them.** Summary tables for each dataset in terms of host and location (continent) is included, as well as in which Figures each dataset is used.

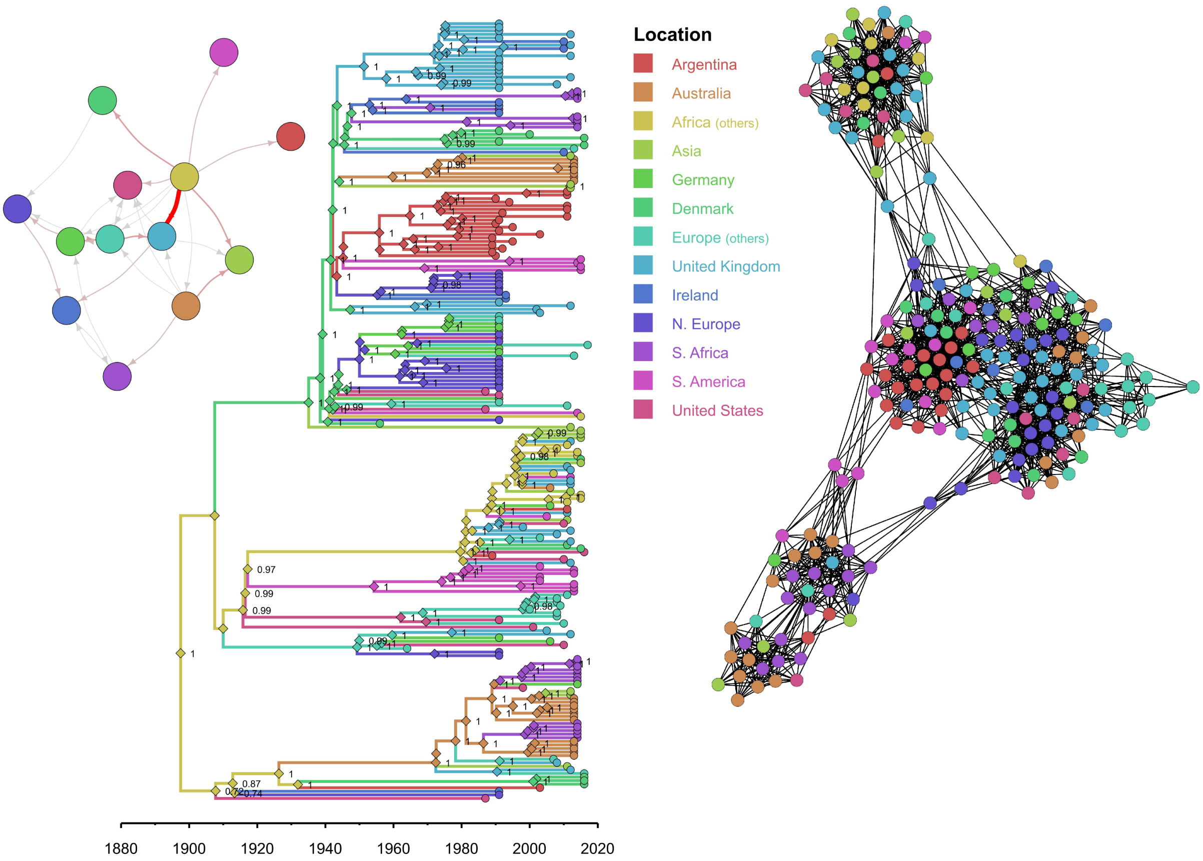

Fig. S2: **Phylogeographic analysis of the multi-host-associated *S. aureus* CC97 based on core and accessory genome**. Bayesian time-stamped tree from a core genome alignment (1,936,889bp, of which were 24,458 variable sites) of CC97 genome sequences, with branches coloured according to the reconstructed location in the discrete trait analysis (left); and network or accessory genome of the same sequences and colours (right, based on 1,221 accessory genes defined as genes in more than 1 genome, and not in all genomes). Inset next to the tree: graphic summary of migrations between countries, in which the thickness and colour (grey->red) of arrows is proportional to the number of migration events inferred.

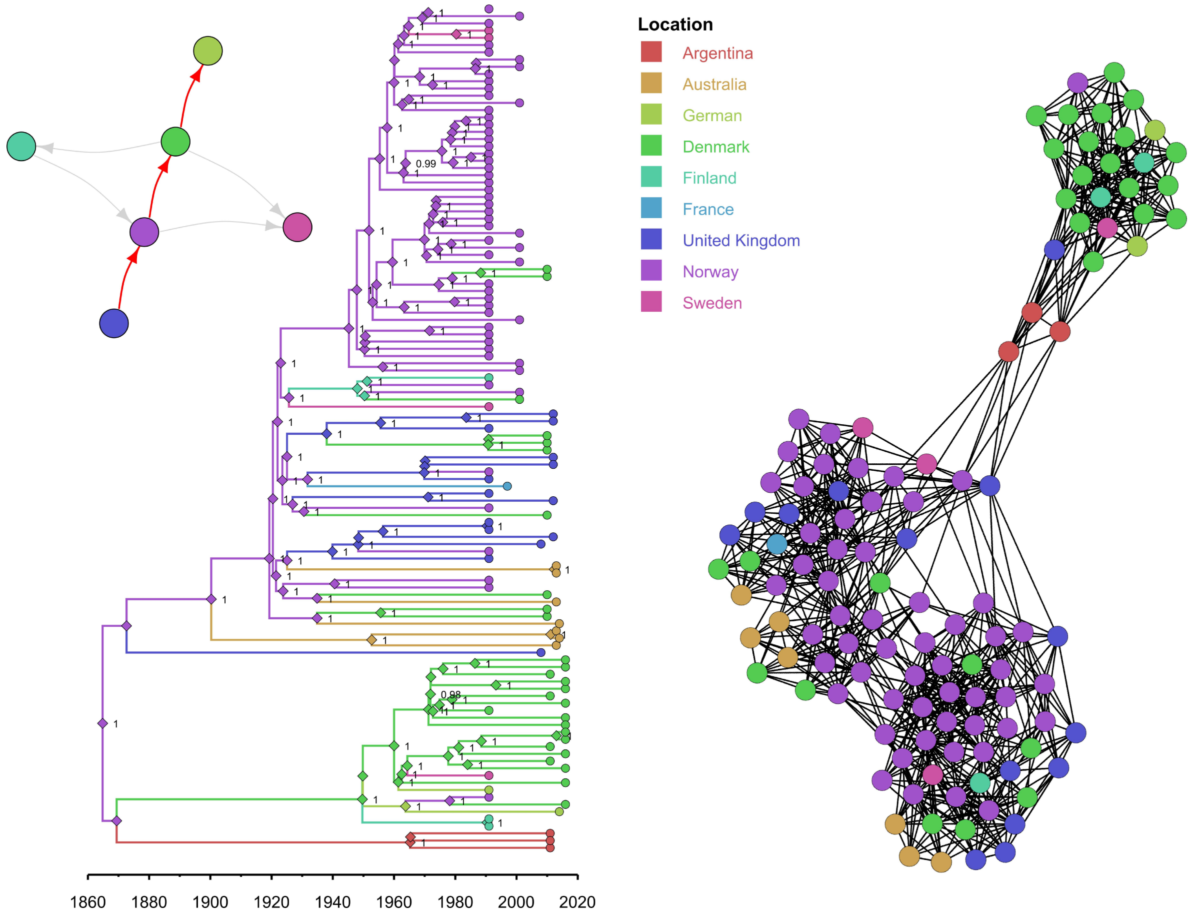

Fig. S3: **Phylogeographic analysis of the multi-host-associated *S. aureus* CC133 based on core and accessory genome**. Bayesian time-stamped tree from a core genome alignment (2,363,270bp, of which 14,435bp were variable sites) of CC133 genome sequences, with branches coloured according to the reconstructed location in the discrete trait analysis (left); and network or accessory genome of the same sequences and colours (right, based on 604 accessory genes defined as genes in more than 1 genome, and not in all genomes). Inset next to the tree: graphic summary of migrations between countries, in which the thickness and colour (grey->red) of arrows is proportional to the number of migration events inferred.

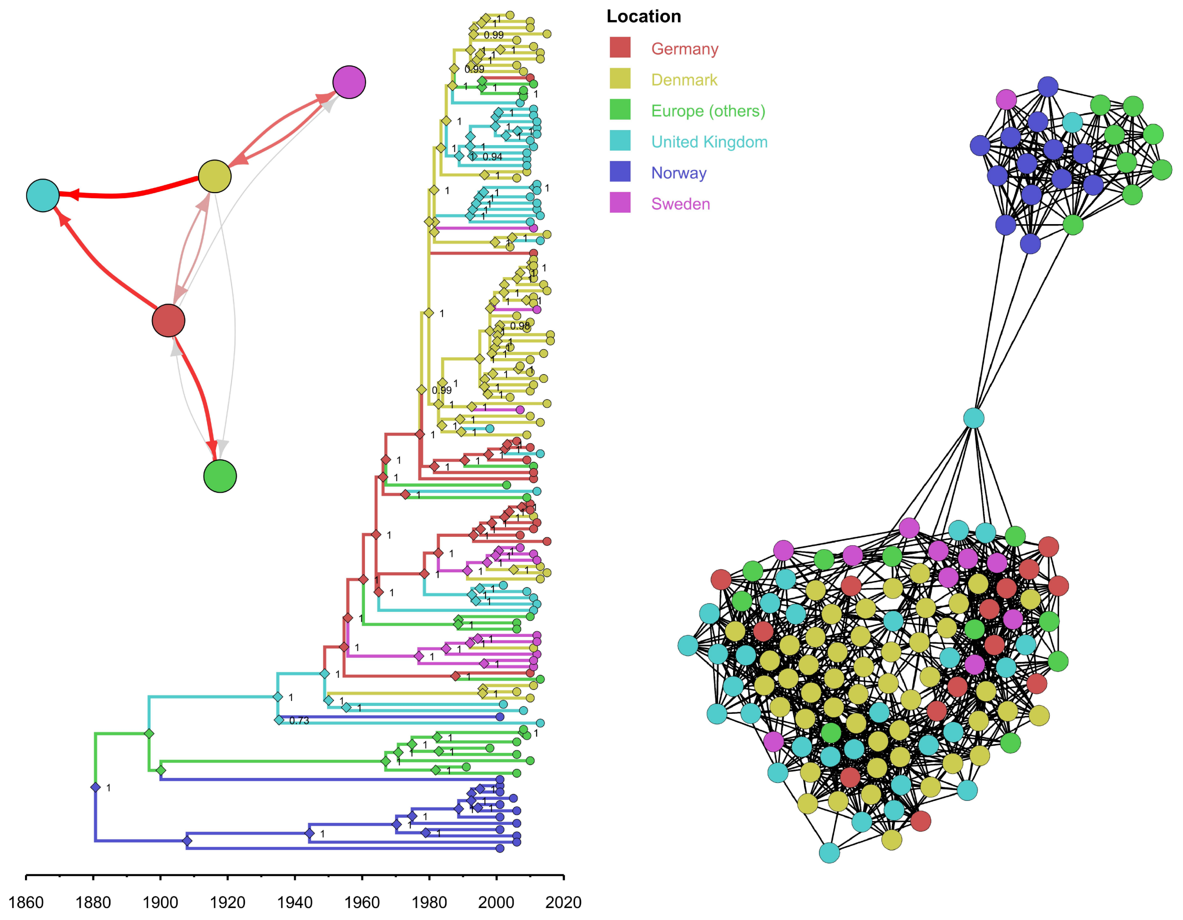

Fig. S4: **Phylogeographic analysis of the multi-host-associated *S. aureus* CC130 based on core and accessory genome**. Bayesian time-stamped tree from a core genome alignment (2,368,277bp, of which 15,123bp were variable sites) of CC130 genome sequences, with branches coloured according to the reconstructed location in the discrete trait analysis (left); and network or accessory genome of the same sequences and colours (right, based on 1,042 accessory genes defined as genes in more than 1 genome, and not in all genomes). Inset next to the tree: graphic summary of migrations between countries, in which the thickness and colour (grey->red) of arrows is proportional to the number of migration events inferred.

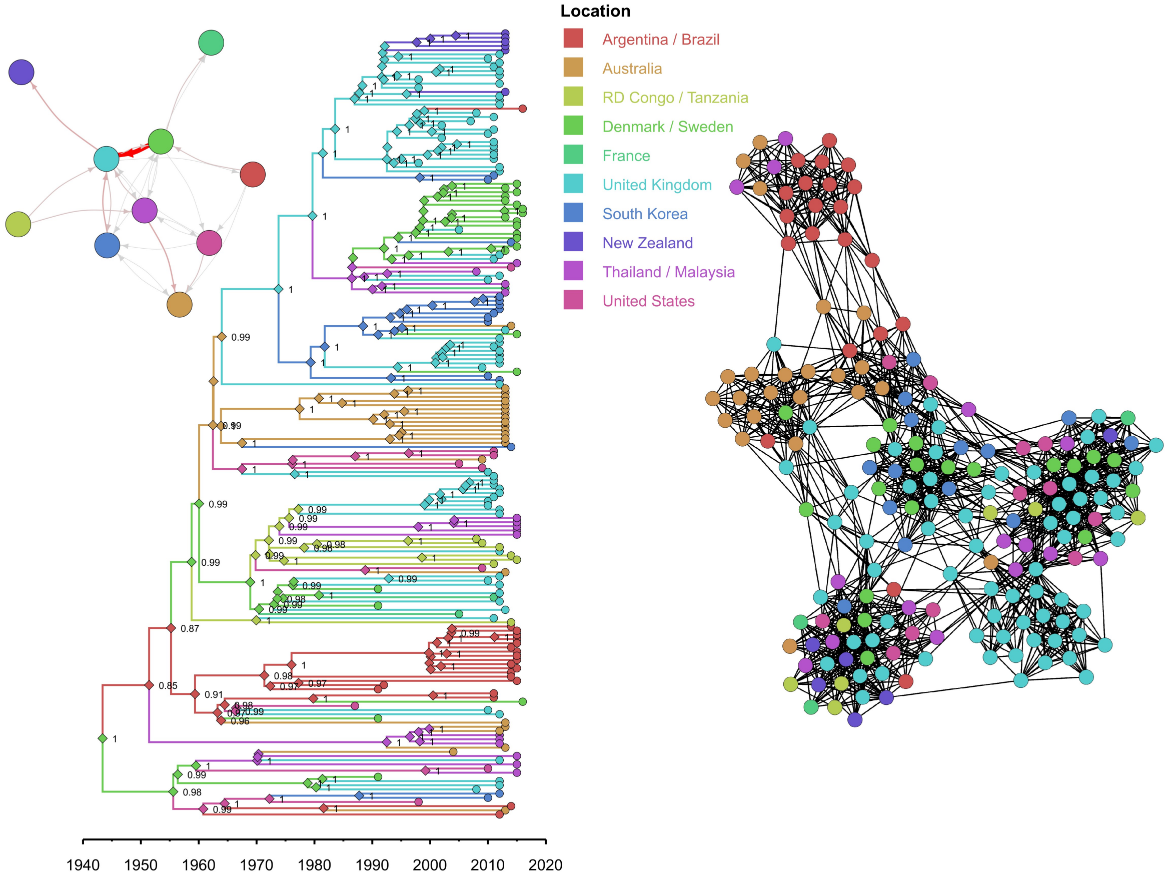

Fig. S5: **Phylogeographic analysis of the multi-host-associated *S. aureus* CC1 based on core and accessory genome**. Bayesian time-stamped tree from a core genome alignment (1,930,409bp, of which 18,984bp were variable sites) of CC1 genome sequences, with branches coloured according to the reconstructed location in the discrete trait analysis (left); and network or accessory genome of the same sequences and colours (right, based on 1,186 accessory genes defined as genes in more than 1 genome, and not in all genomes). Inset next to the tree: graphic summary of migrations between countries, in which the thickness and colour (grey->red) of arrows is proportional to the number of migration events inferred.

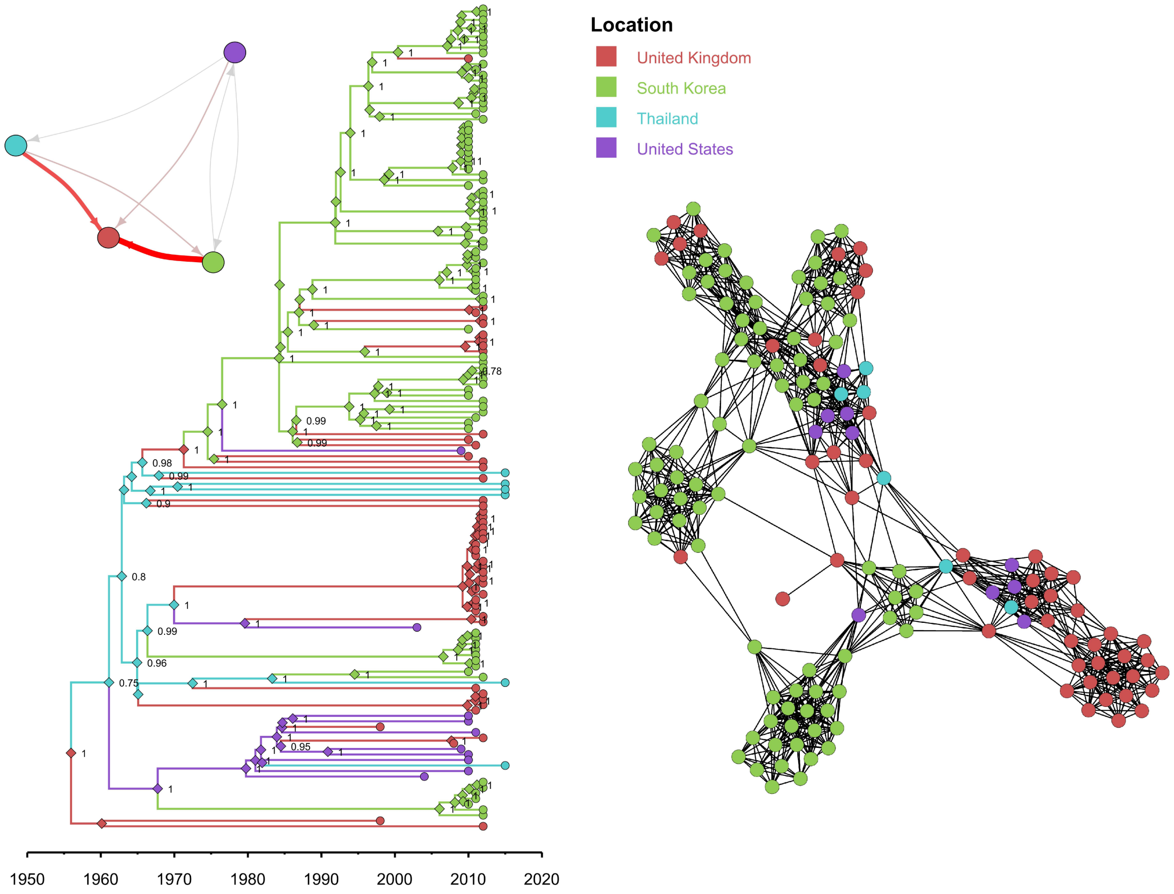

Fig. S6: **Phylogeographic analysis of the multi-host-associated *S. aureus* CC188 based on core and accessory genome**. Bayesian time-stamped tree from a core genome alignment (2,451,373bp, of which 7,378bp were variable sites) of CC188 genome sequences, with branches coloured according to the reconstructed location in the discrete trait analysis (left); and network or accessory genome of the same sequences and colours (right, based on 717 accessory genes defined as genes in more than 1 genome, and not in all genomes). Inset next to the tree: graphic summary of migrations between countries, in which the thickness and colour (grey->red) of arrows is proportional to the number of migration events inferred.

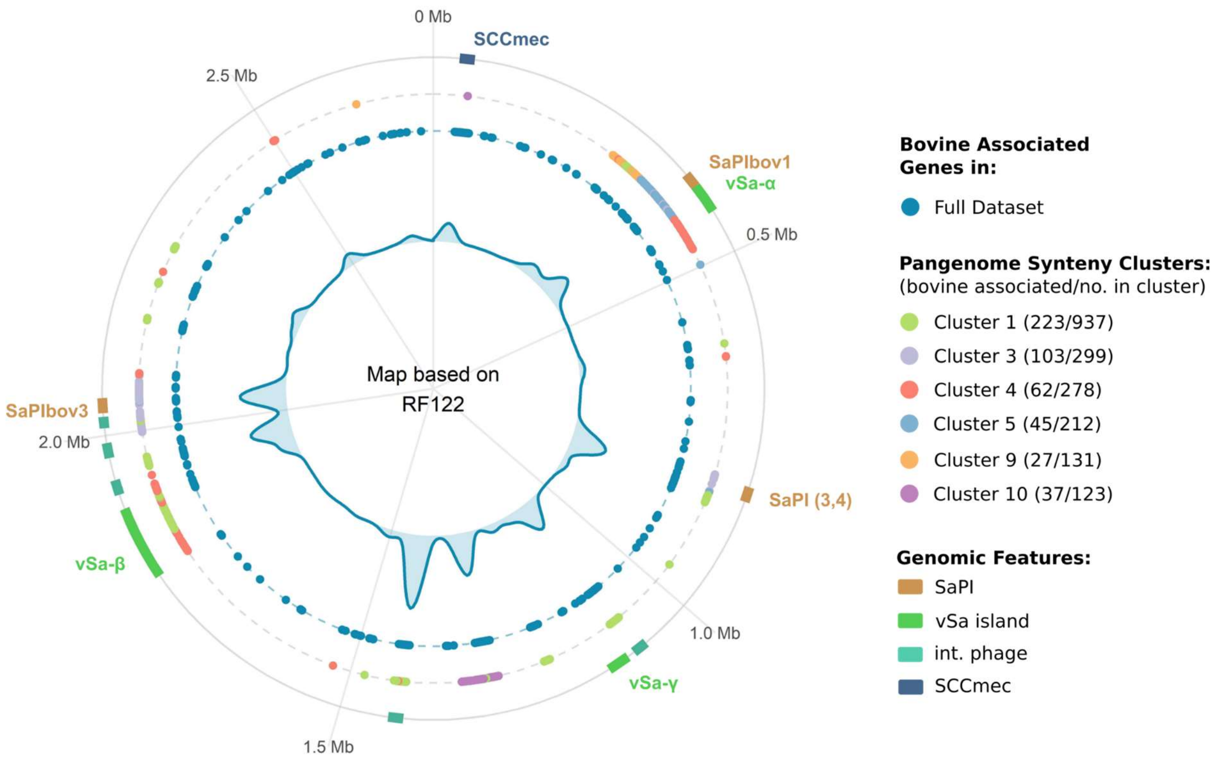

Fig. S7. Pangenome synteny clusters significantly enriched in bovine *S. aureus* associated genes, mapped to the reference genome RF122. Central density plot displays distribution of bovine-associated genes. Circle dot plots represent the location of bovine associated genes (inner), pangenome synteny clusters (middle), and known genomic features of interest (outer).

Supplementary Tables

Table S1. Distribution of inferred host changes in the global bovine-enriched *S. aureus* phylogeny from the SIMMAP analyses.

State change	Median number of changes (95%HPD)
Others -> Human	277.5 (251-313)
Human -> Others	188.5 (159-208)
Human -> Bovine	182 (160-202)
Bovine -> Others	131.5 (114-149)
Others -> Bovine	114 (95-133)
Bovine -> Human	63.5 (46-77)

Table S2. Distribution of inferred location changes (i.e. migrations) in the global bovine-enriched *S. aureus* phylogeny from the SIMMAP analyses. The table shows only those with median ≥10 changes for simplicity.

**State change**	**Median number of changes (95%HPD)**
N America → SS Africa	41 (30-52)
Ireland → Switzerland	32.5 (23-44)
N America → Ireland	31 (22-38)
UK → Ireland	28 (20-36)
N America → Switzerland	27 (18-36)
Ireland → N America	26.5 (18-35)
Switzerland → N America	26 (14-34)
Australia → SE Asia	24 (13-32)
Denmark → Sweden	19 (11-30)
S America → Sweden	15 (8-25)
Germany → N America	13 (8-19)
Denmark → Norway	13 (6-19)
UK → Denmark	13 (9-18)
Germany → Switzerland	12 (7-18)
Germany → Denmark	12 (6-17)
N America → S Europe	11 (6-19)
Sweden → Switzerland	11 (4-18)
Sweden → S Europe	11 (6-17)
Switzerland → Ireland	10 (5-17)
N America → Germany	10 (5-17)
Norway → UK	10 (5-15)
Sweden → Finland	10 (5-15)

Table S3. Analysis of Association Index ratio of phylogenetic distribution and trait (host, location, clustering based on accessory genome) performed with BaTS. The AI ratio ranges from 0 (perfect association) to 1 (no association).

Clonal Complex	# Hosts	Host AI	# Locations	Location AI	# of MCL Clusters (*)	MCL AI
**CC151**	-	n/a	11	0.13 (0.13-0.14)	5	0.42 (0.42-0.44)
**CC97**	2	0.33 (0.29-0.36)	13	0.35 (0.34-0.36)	5	0.21 (0.21-0.22)
**CC1**	2	0.25 (0.24-0.28)	10	0.37 (0.37-0.37)	5	0.59 (0.57-0.61)
**CC188**	2	0.04 (0.04-0.05)	4	0.1 (0.09-0.11)	5	0.26 (0.26-0.26)
**CC133**	2	0.39 (0.31-0.49)	9	0.24 (0.24-0.25)	3	0.51 (0.48-0.52)
**CC130**	3	0.67 (0.58-0.79)	6	0.20 (0.20-0.20)	3	0.34 (0.31-0.38)
**CC425**	2	0.26 (0.19-0.38)	-	n/a	2	0.41 (0.35-0.51)

(*) Clusters of accessory genomes defined using i = 1.40

Table S4: Goodman-Kruskal tau (GKτ) values for association between accessory genome clusters and host/location. The values range from 0 (no predictability) to 1 (full predictability), i.e. the higher the value the better clustering matches/predicts the metadata variable.

**Clonal Complex**	**Cluster threshold (MCLi)**	**# clusters**	**# hosts**	**#locations**	**G-Kτ Host**	**G-Kτ Location**
**CC1**	1.40	5	2	14	0.569	0.163
	2.10	7	2	14	0.579	0.211
**CC97**	1.40	5	3	13	0.607	0.086
	2.00	6	3	13	0.611	0.097
**CC130**	1.40	3	3	7	0.275	0.138
	2.10	4	3	7	0.282	0.143
**CC133**	1.40	3	2	9	0.066	0.169
	2.10	4	2	9	0.150	0.18
**CC151**	1.40	8	1	11	n/a	0.312
	2.10	11	1	11	n/a	0.418
**CC188**	1.40	5	2	4	0.355	0.310
	2.10	8	2	4	0.565	0.435
**CC425**	1.40	2	2	4	0.000	0.012
	2.10	5	2	4	0.112	0.605

Table S5: Distribution of the *mecA* gene among selected Clonal Complexes (CCs) and Host species

	**Bovine**	**Human**	**Swine**	**Ovine**	**Other**	**Total**
**CC398**	58/82 (70.73%)	332/417 (79.62%)	55/91 (60.44%)	na	46/70 (65.71%)	491/660 (74.39%)
**CC5**	10/26 (38.46%)	229/326 (70.25%)	35/45 (77.78%)	na	0/57 (0%)	274/408 (67.16%)
**CC8**	10/38 (26.32%)	254/363 (69.97%)	na	na	10/20 (50%)	274/421 (65.08%)
**CC45**	55/69 (79.71%)	10/62 (16.13%)	na	na	0/3 (0%)	65/134 (48.51%)
**CC1**	1/119 (0.84%)	39/139 (28.06%)	0/2 (0%)	na	1/36 (2.78%)	41/296 (13.85%)
**CC97**	20/624 (3.21%)	6/63 (9.52%)	4/8 (50%)	na	4/7 (57.14%)	34/702 (4.84%)
**CC130**	1/114 (0.88%)	0/93 (0%)	0/16 (0%)	0/24 (0%)	0/9 (0%)	1/256 (0.39%)
**CC133**	0/106 (0%)	0/1 (0%)	na	1/19 (5.26%)	0/4 (0%)	1/130 (0.77%)
**CC151**	0/276 (0%)	na	na	na	na	0/276 (0%)
**CC188**	0/92 (0%)	1/66 (1.52%)	1/1 (100%)	na	0/8 (0%)	2/167 (1.20%)
**CC425**	1/126 (0.79%)	0/15 (0%)	na	na	0/2 (0%)	1/143 (0.70%)
**Other**	21/324 (6.48%)	245/664 (36.90%)	11/57 (19.30%)	na	4/203 (1.97%)	281/1,248 (22.52%)
**Total**	177/1,996 (8.87%)	1,116/2,209 (50.52%)	106/220 (48.18%)	1/43 (2.33%)	65/373 (17.43%)	1,465/4,841 (30.26%)

Table S6: Distribution of the *mecC* gene among selected Clonal Complexes (CCs) and Host species

	Bovine	Human	Swine	Ovine	Other	Total
**CC130**	109/114 (95.61%)	92/93 (98.92%)	16/16 (100%)	6/24 (25%)	2/36 (22.22%)	225/256 (87.89%)
**CC425**	101/126 (80.16%)	14/15 (93.33%)	na	na	0/2 (0%)	115/143 (80.42%)
**CC133**	1/106 (0.94%)	0/1 (0%)	na	1/19 (5.26%)	0/4 (0%)	2/130 (1.54%)
**CC151**	1/276 (0.36%)	na	na	na	na	1/276 (0.36%)
**CC1**	1/119 (0.84%)	0/139 (0%)	0/2 (0%)	na	0/36 (0%)	1/296 (0.34%)
**Other**	3/1,255 (0.24%)	1/1,888 (0.05%)	0/202 (0%)	na	1/295 (0.34%)	6/3,740 (0.36%)
**Total**	216/1,996 (10.82%)	107/2,209 (4.84%)	16/220 (7.27%)	7/43 (16.28%)	3/373 (0.80%)	349/4,841 (7.21%)
